# Access Denied

**DOI:** 10.1289/ehp.10729

**Published:** 2007-10

**Authors:** Thomas J. Goehl

**Affiliations:** Publishing Consultant, Morrisville, North Carolina, E-mail: tjgoehl@yahoo.com

Accessibility to state-of-the-art environmental health studies, public health research, and clinical medical practices is a major problem in developing countries. To compound the problem, the information found in international journals is often not relevant to the developing world (Richards 2004). National and regional journals attempt to fill the void by publishing robust and relevant information. However, these journals face difficulties in sustainability and local access (Ofori-Adjai 2006).

There are many reasons for the inaccessibility to both information coming out of the developed world and the developing world itself. For example, subscriptions to international journals often present a financial barrier, whereas regional journals often are poorly supported and face difficult distribution problems.

How can developing countries improve the health of their populations when they are effectively removed from the stream of knowledge from the developed world and their own region? The solutions include free access to the world’s literature on health care and assistance in developing their research capabilities, publication processes, and distribution methods.

Why should the developed world care about this issue? There is a moral imperative: I consider access to information to facilitate adequate health care a human right. An immediate impact of accessibility would be improvements in local clinical practice. In the long term, policy makers would be able to use knowledge-based decision making in formulating laws to help improve public health in their countries.

There are global benefits for disseminating information in and from developing countries (Hryhorczuk 2006). First, because diseases and pollution are not constrained by geographic or political boundaries, the developed world would gain from, for example, improved access to information on infectious diseases that could affect their populations. Second, in regard to pollutants, conditions in developing countries often provide research opportunities that benefit both developed and developing countries. Finally, sharing information is good foreign policy for developed nations.

Major steps have been taken to overcome some of the problems associated with improving the developing world’s access to information available in international journals. For example, open access has been a growing trend within the publication industry. Many journals now have open access policies, including *EHP* (Goehl and Olden 2004), and others have embraced the spirit of open access but not fully implemented all of its provisions (i.e., free and immediate access to journal articles, as well as placing articles in a major digital archive).

Many new open-access journals have been established in the last several years, including the *Public Library of Science* journals (PLoS 2007). All content of PLoS journals may be redistributed and reused according to the terms of the Creative Commons Attribution License (Creative Commons 2007).

Digital archives play a key role in facilitating access to the world’s literature. One of the major digital archives is PubMed Central (2007), a National Institutes of Health archive that provides a free digital archive of biomedical and life sciences literature. Three other digital archives that deserve mention are Bioline International, Scientific Electronic Library Online (SCIELO 2007), and African Journals Online (AJOL 2007). These archives were established to promote the global exchange of scientific knowledge by providing access to peer-reviewed journals published in developing countries.

Journal indexes such as PubMed (2007) and MEDLINE (available through PubMed) are critical to finding scientific and medical information. Access to Latin American literature has been enhanced by Literatura Latino Americana e do Caribe em Ciências da Saúde (LILACS 2007). This database registers the health scientific–technical literature published by Latin American and Caribbean authors, which is frequently absent from the international databases.

Because of the paucity of relevant research and medical information coming out of the developed world, there is a unique niche for regional journals that publish research relevant to local situations. These journals are also a resource for continuing education and a forum for aspiring researchers to enhance research and publication skills.

There are major obstacles to regional journal development, starting with insufficient resources. Research papers are often lacking in rigor, and the quality of writing is often a problem. The review process is often limited by the number of experts fluent in the local language. Also, some authors are reluctant to submit quality papers to local journals because of concern about the journal’s stature and the limited dissemination of research results.

To address these issues, national and regional journals in developing countries must be made more attractive to researchers by enhancing the quality of the publications. Institutions such as the World Health Organization, the Pan American Health Organization, the Fogarty International Center (FIC), the National Library of Medicine (NLM), and private philanthropic foundations have provided support, but much more support is needed.

A successful model for journal capacity building is the African Journal Partnership (AJP) project that was initiated in 2004 by the FIC, the NLM, and the National Institute of Environmental Health Sciences and managed by the Council of Science Editors (Tillett 2005). These organizations helped establish committed journal-to-journal partnerships of international journals and African medical journals. Through this partnership, editors have received training to improve sustainability and publishing regularity of the journals; writing workshops have been sponsored to improve the quality of the submitted manuscripts; peer reviewers have been trained to enhance the peer-review process; and journal visibility has been enhanced by establishing or improving websites and by fostering the acceptance into international indexes (Sidibe 2007). Currently two of the four African journal partners (*Mali Médical* and *African Health Sciences*) are indexed in MEDLINE/PubMed.

The AJP is helping to address the accessibility issue to national and regional journals in Africa. To address similar issues in Latin America, a task group of the Committee on Data for Science and Technology (CODATA 2007), a committee of the International Council for Science, recently convened a workshop, “Strategies for Permanent Access to Scientific Information in Latin America.” During that meeting, a proposal was presented to develop a partnership similar to the AJP that would promote partnerships between high-ranking international environmental, medical, and public health journals with journals in Latin American and Caribbean countries. The focus again will be on one-on-one efforts (Goehl 2007). Considerable interest was expressed in this proposal, and a search is under way for governmental and nongovernmental organizations to provide the infrastructure to facilitate the necessary efforts.

Although accessibility to state-of-the-art environmental health studies, public health research, and clinical information continues to be a major problem in the developing world, the open-access initiatives and efforts to help build sustainable national and regional journals offer significant hope for overcoming this problem.

## Figures and Tables

**Figure:**
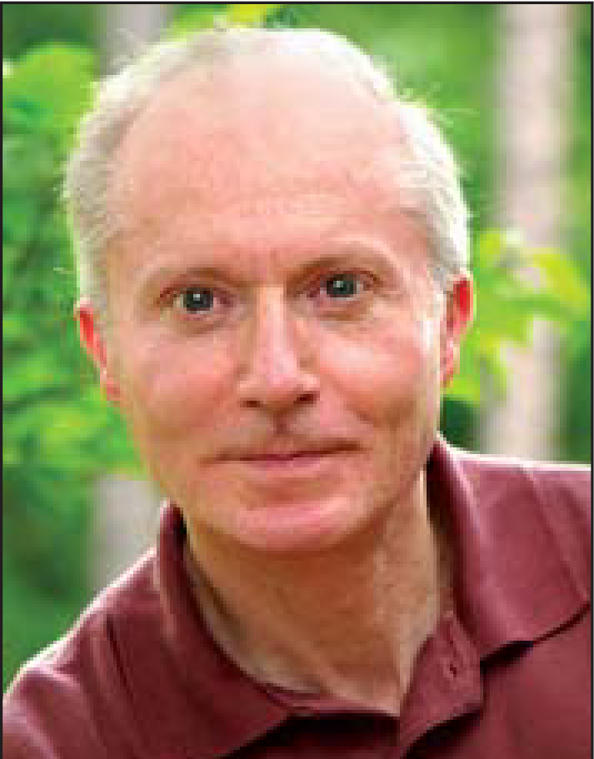
Thomas J. Goehl
